# Elevated Plasma Levels of sIL-2R in Complex Regional Pain Syndrome: A Pathogenic Role for T-Lymphocytes?

**DOI:** 10.1155/2017/2764261

**Published:** 2017-05-28

**Authors:** Krishna D. Bharwani, Maaike Dirckx, Dirk L. Stronks, Willem A. Dik, Marco W. J. Schreurs, Frank J. P. M. Huygen

**Affiliations:** ^1^Center for Pain Medicine, Erasmus MC University Medical Center Rotterdam, Rotterdam, Netherlands; ^2^Department of Immunology, Laboratory Medical Immunology, Erasmus MC University Medical Center Rotterdam, Rotterdam, Netherlands

## Abstract

The immune system has long been thought to be involved in the pathophysiology of complex regional pain syndrome (CRPS). However, not much is known about the role of the immune system and specifically T-cells in the onset and maintenance of this disease. In this study, we aimed to evaluate T-cell activity in CRPS by comparing blood soluble interleukin-2 receptor (sIL-2R) levels between CRPS patients and healthy controls. CRPS patients had statistically significant elevated levels of sIL-2R as compared to healthy controls (median sIL-2R levels: 4151 pg/ml (Q3 − Q1 = 5731 pg/ml − 3546 pg/ml) versus 1907 pg/ml (Q3 − Q1: 2206 pg/ml − 1374 pg/ml), *p* < 0.001, resp.). Furthermore, sIL-2R level seems to be a good discriminator between CRPS patients and healthy controls with a high sensitivity (90%) and specificity (89.5%). Our finding indicates increased T-cell activity in patients with CRPS. This finding is of considerable relevance as it could point towards a T-cell-mediated inflammatory process in this disease. This could pave the way for new anti-inflammatory therapies in the treatment of CRPS. Furthermore, sIL-2R could be a promising new marker for determining inflammatory disease activity in CRPS.

## 1. Introduction

Complex regional pain syndrome (CRPS) is a clinical disorder characterized by severe pain in an affected extremity that is accompanied by sensory, motor, vasomotor, and sudomotor disturbances [1]. CRPS is often preceded by an injury to an extremity such as a fracture or surgery [2].

The exact pathophysiology of CRPS is still unknown. CRPS is considered to be a multimechanism disease [3]. The following mechanisms have been proposed to play a role in CRPS: inflammation, central and peripheral sensitization, altered sympathetic nervous system function, endothelial dysfunction, brain plasticity, and psychological factors [3–5].

Inflammation as an underlying pathogenic mechanism for CRPS has long been a topic of debate, as systemic markers of inflammation such as erythrocyte sedimentation rate (ESR), c-reactive protein (CRP), and white blood cell (WBC) count are usually not elevated in CRPS patients [6–8]. However, classic signs of inflammation such as pain, swelling, and redness are often present during physical examination, especially in the initial stages of the disease, suggesting that inflammation does contribute to CRPS [9, 10]. The latter is supported by research findings that demonstrated increased levels of the proinflammatory cytokines' tumor necrosis factor alpha (TNF-*α*) and interleukin- (IL-) 6 in skin blister fluid of the affected limbs versus the unaffected limbs of CRPS patients [11, 12]. Further, recent studies have confirmed evidence of systemic inflammation in the venous blood of CRPS patients [7, 13].

An interesting theory on inflammation in CRPS was put forward by Goebel and Blaes suggesting that CRPS is a novel kind of antibody-mediated autoimmune disease [14].

To expand on this theory, our research group previously analyzed the presence of antinuclear antibodies (ANA) and antineuronal antibodies in CRPS patients and demonstrated increased ANA positivity in CRPS patients as compared to healthy controls (33% versus 4%, resp., *p* < 0.001) [15]. The frequency of positivity for antineuronal antibodies did not differ between the groups [15]. Considering our findings and those of Goebel et al., we could not define CRPS as an antibody-mediated autoimmune disease in accordance with Witebsky's criteria for autoimmune diseases [14–19]. Therefore, we shifted our focus to exploring the role of T-cells in the inflammation seen in CRPS.

Hitherto, very few studies have been conducted on the role of T-cells in CRPS [6, 20, 21]. These studies were conducted using different methodologies and had different outcome parameters, making comparison between the studies and thus establishment of a firm conclusion difficult. Furthermore, some data presented in these studies are contradictory; for example, while one study showed no difference in blood lymphocyte populations (i.e., (cytotoxic) CD8+ T-cells, CD4+ T-cells, B-cells, and natural killer (NK) cells) between CRPS patients and healthy controls, another study found that CRPS was associated with a significant reduction in the number of CD8+ T-cells [6, 20].

Another approach to study T-cell involvement in CRPS is to analyze whether there are indications of increased T-cell activity in CRPS, for instance by the measurement of soluble interleukin-2 receptor levels in the peripheral blood.

Interleukin-2 (IL-2) is a cytokine crucially important in regulating activation, proliferation, and survival of different T-cell subsets [22, 23]. This effect of IL-2 is mediated through the IL-2 receptor which consists of the common *γ*-chain (CD132), a *β*-chain (CD122), and an *α*-chain (CD25) [22, 24]. CD25 is strongly expressed on activated T-cells which also secrete this molecule as a soluble variant (referred to as soluble IL-2 receptor; sIL-2R) from the cell membrane into the circulation [22, 25, 26].

Peripheral blood levels of sIL-2R have been found to reflect the level of T-cell activation, and elevated sIL-2R levels correlate with disease activity in for instance rheumatoid arthritis and sarcoidosis, diseases in which T-cell activation is centrally involved [22, 24, 25, 27–30].

Finding increased levels of sIL-2R in CRPS could be indicative of a T-cell-mediated inflammatory process in this disease. This finding could contribute to a better understanding of the underlying inflammatory pathophysiological mechanism in CRPS. This could further lead to the development and application of (new) therapies in the treatment of CRPS patients with (T-cell-mediated) inflammation as an underlying mechanism of their disease.

Therefore, the aim of this study was to evaluate T-cell activity in CRPS by examining the levels of sIL-2R in a group of CRPS patients and comparing these to sIL-2R levels in a group of healthy controls.

## 2. Materials and Methods

### 2.1. Ethical Approval

This study was approved by the Medical Ethics Committee of Erasmus MC University Medical Center Rotterdam (MEC-2016-172).

### 2.2. Patients and Controls

Patients who visited the Center for Pain Medicine at Erasmus MC University Medical Center Rotterdam between 2001 and 2007 and fulfilled the Harden-Bruehl diagnostic criteria for CRPS were invited to participate in various ongoing studies [31].

In the context of these studies, venous blood samples were drawn from patients and plasma was stored in a refrigerator at −80 degrees Celsius for use in future research with permission from the patients.

For this study, we examined the levels of sIL-2R in the plasma of 80 adult patients with CRPS type I and compared this to sIL-2R levels measured in 76 anonymous healthy blood bank donors who also had given permission to use their blood for future research purposes.

### 2.3. sIL-2R Analysis

Venous blood samples were centrifuged at 3000 rpm immediately after collection for a duration of 10 minutes. Plasma was stored at −80 degrees Celsius and thawed to room temperature for sIL-2R analysis.

sIL-2R plasma levels were quantified using an enzyme-linked immunosorbent assay (Human sCD25/sIL-2R ELISA kit, Besancon, Cedex, France) in accordance with the manufacturer's instructions. sIL-2R levels are expressed in picograms per milliliter (pg/ml), and levels > 2500 pg/ml are considered elevated.

### 2.4. Statistical Analysis

Descriptive statistics were used to determine the frequencies of the demographic variables and plasma sIL-2R levels and to describe the measures of central tendency and of variability. The Shapiro-Wilk test was used to test the distribution of these variables for normality. Results are reported in medians and interquartile ranges (Q3 − Q1) if the distribution is skewed and otherwise in means and standard deviations (SD).

Differences in sIL-2R levels between the CRPS patients and the healthy blood bank donors were analyzed using the independent-samples Mann–Whitney *U* test (two-sided).

A possible association between sIL-2R levels of the CRPS patients, their age, gender, and duration of the CRPS was also explored. An association with gender was evaluated using the independent-samples Mann–Whitney *U* test (two-sided). With regard to the age of patients and the duration of CRPS, Spearman's rank correlation was used (two-sided).

A binary logistic regression was used to evaluate the contribution of the level of sIL-2R to the prediction of the group (CRPS patients versus healthy controls). A receiver operating characteristic (ROC) curve was computed. The sensitivity, specificity, positive predictive value (PPV), and negative predictive value (NPV) of sIL-2R were calculated.

The alpha level for statistical significance was set at 0.05. Analyses were performed using IBM SPSS Statistics 21.

## 3. Results

Plasma was available from 80 CRPS patients. The characteristics of our CRPS patient group are depicted in Table 1.

**Figure 1:**
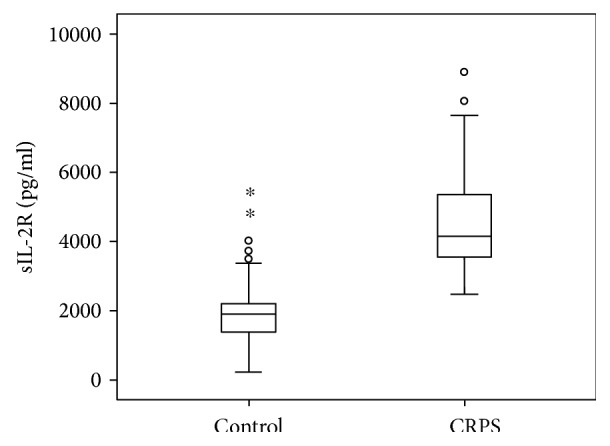
Boxplot of the sIL-2R level by the experimental group. ^∗^Healthy controls with sIL-2R levels that are more than three times the IQ-range.

The median sIL-2R level of the CRPS group was statistically significantly higher compared to the median sIL-2R level of the control group. The median of the CRPS patients was 4151 pg/ml (Q3 − Q1 = 5731 pg/ml − 3546 pg/ml) and that of the control subjects was 1907 pg/ml (Q3 − Q1: 2206 pg/ml − 1374 pg/ml), *p* < 0.001 (see Figure 1).

**Figure 2:**
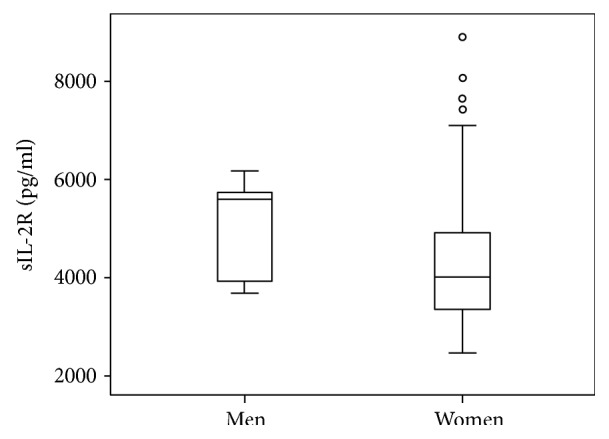
Boxplot of the sIL-2R level by gender in CRPS patients.

Plasma levels of sIL-2R between male and female CRPS patients were also compared. The sIL-2R plasma levels in men were statistically significantly higher than in women (men 5602 pg/ml, Q3 − Q1 = 5829 pg/ml − 3921 pg/ml versus women 4016 pg/ml, Q3 − Q1 = 4951 pg/ml − 3286 pg/ml, *p* = 0.03) (see Figure 2).

No association was found in the CRPS patient group between sIL-2R levels and the duration of disease (*r*_s_ = −0.18, *p* = 0.10) nor between sIL-2R levels and the age of patients (*r*_s_ = 0.12, *p* = 0.28).

**Figure 3:**
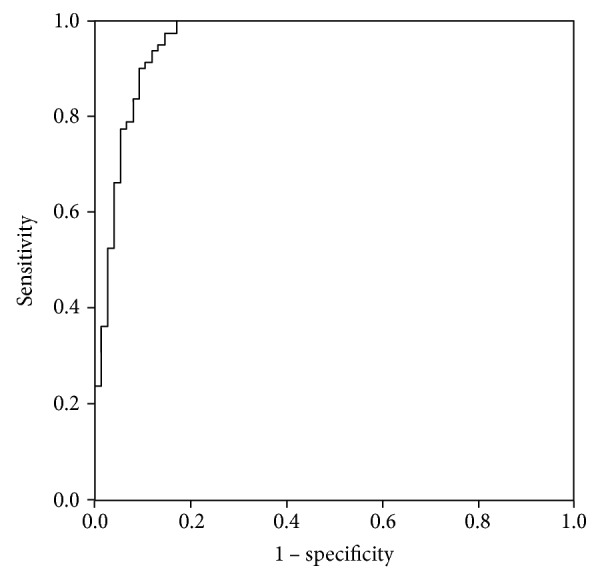
Receiver operating characteristic curve of sIL-2R as a predictor of group (CRPS patients versus healthy controls). Area under the curve 0.958 (SE 0.016), *p* < 0.001.

The level of sIL-2R showed a favorable discrimination between CRPS patients and healthy controls. The sensitivity was observed to be 90%, specificity 89.5%, PPV 90%, and NPV 89.5%, using a cut-off value of 0.5, which corresponds with an sIL-2R level of 3730 pg/ml (see Table 2, Figure 3).

## 4. Discussion

This study was conducted to explore whether T-cell activation is involved in the pathophysiology of CRPS. To this end, venous blood levels of sIL-2R in CRPS patients were compared to those of healthy controls. Our data clearly demonstrate that plasma sIL-2R levels are significantly elevated in CRPS patients. This finding is of considerable relevance as it indicates increased T-cell activity in CRPS and therefore could point towards an underlying T-cell-mediated inflammatory process in this disease.

There is one earlier study which has shown significantly elevated plasma levels of sIL-2R in CRPS patients [32]. However, the aim of this study was to conduct an explorative analysis of various plasma analytes in CRPS patients and to subsequently derive different CRPS clusters based on their findings [32]. The authors did not relate this finding of higher plasma levels of sIL-2R to T-cell activity [32]. This seems to be more an ancillary finding, but it strongly supports our results.

In our study, we observed higher sIL-2R plasma levels in men with CRPS as compared to those in women with CRPS. We could not compare the gender distribution of sIL-2R levels in our CRPS group to the group of healthy controls as this was an anonymous group without any available demographic data. However, other studies do not report elevated blood sIL-2R levels in healthy men compared to healthy women [22, 33–35].

Our finding of a significant difference in sIL-2R levels between CRPS men and women conflicts with the finding of the previously mentioned explorative study [32]. This study found no differences in sIL-2R levels between men and women with CRPS [32]. One explanation for the contradictory results could be the possibility of higher disease severity in our group of male CRPS patients, as sIL-2R levels have been shown to correlate with disease severity in other disease entities [28].

We found no association between sIL-2R levels and age in the CRPS group. Studies in healthy individuals have shown sIL-2R levels to vary with age [22]. Children (age 1–14 years) and the elderly (age 67–99 years) have been shown to have higher sIL-2R levels as compared to (young) adults (age 22–67 years) [22, 33, 35]. This could explain why no association was found between sIL-2R levels and age as our CRPS patient sample consisted mainly of (young) adults with a mean age of 44.4 years (SD 12.25).

No association was found between sIL-2R levels and duration of disease, suggesting immune system activation throughout the entire disease course in a subgroup of CRPS patients.

Finally, our findings show sIL-2R level to be a good discriminator between CRPS patients and healthy controls. This finding could lead not only to the use of sIL-2R as a marker of inflammatory disease activity in CRPS but also to the use of sIL-2R during the diagnostic work-up of this disease. As per our knowledge, no study has yet been performed on this subject. Consequently, further studies are required to validate sIL-2R as a marker of inflammatory disease activity in CRPS and to establish a validated diagnostic cut-off value to differentiate CRPS from other chronic pain diseases (e.g., fibromyalgia) and autoimmune and autoinflammatory disorders (e.g., rheumatoid arthritis).

While our findings indicate increased T-cell activity in CRPS, it is still unclear what subset of T-cells is being activated as sIL-2R is T-cell subset nonspecific and is thus measured during activation of different T-cell subsets [22].

Previous studies have tried characterizing changes in lymphocyte subsets in CRPS [20, 21]. In 2007, Kaufmann et al. investigated the influence of pain and stress on lymphocyte numbers, lymphocyte subpopulations, and T-helper 1/T-helper 2 (Th1/Th2) ratio in CRPS patients, fibromyalgia patients, and healthy controls [20]. They found a significant reduction of CD8+ T-lymphocytes in CRPS patients as compared to healthy controls [20].

In 2015, Osborne et al. studied skin immune cell populations in long-standing CRPS and found no significant differences in overall immune cell infiltrates between CRPS affected and unaffected limbs [21].

As stated previously, while our findings indicate increased T-cell activity in CRPS, we cannot specify which subset(s) of T-cells are active, making it difficult to relate our findings to the findings from the studies mentioned above [20, 21]. Moreover, both studies used different methodologies to study the involvement of T-cells (i.e., skin punch biopsies in the study by Osborne et al. and venous blood samples in the study by Kaufmann et al.) [20, 21]. Furthermore, Osborne et al. compared the unaffected and affected extremities in CRPS patients (side to side comparison) while Kaufmann et al. looked at differences between CRPS patients, fibromyalgia patients, and healthy controls [20, 21]. As a consequence, comparison of our results to those of Kaufmann et al. or to those of Osborne et al. is rather meaningless [20, 21].

Further, the internal and external validity of this current study should be evaluated taking into account the potential incomparability of the experimental groups in terms of age, gender, past history, and medication as demographic data and medical history were unavailable for the control group.

Finally, our study consists of a cross-sectional measurement of sIL-2R levels in CRPS patients and healthy blood bank donors. It would be interesting to prospectively study sIL-2R levels during the course of this disease. This could increase our understanding on the role of T-cell activity in the onset and maintenance of CRPS.

Based on the findings of this study, we propose a role of T-cell-mediated inflammation as an underlying mechanism in the pathophysiology of CRPS.

Our results are of considerable relevance as the involvement of T-cells in CRPS could lead to a better understanding of the rather complex pathophysiology of this disease. The findings of this study and the results of possible future research might lead to new therapeutic targets in the treatment of CRPS patients with (T-cell-mediated) inflammation as an underlying mechanism of disease, thereby paving the way for new anti-inflammatory and/or immunomodulating therapies in the management of CRPS.

## 5. Conclusion

The median sIL-2R level of CRPS patients was found to be significantly increased as compared to that of a group of healthy blood bank donors. This result indicates increased T-cell activity in CRPS.

This finding could point towards a T-cell-mediated inflammatory process in CRPS, which could pave the way for new anti-inflammatory therapies in the treatment of CRPS patients with (T-cell-mediated) inflammation as their underlying mechanism of disease. However, the precise role of T-cells in the pathophysiology of CRPS has yet to be unraveled.

Furthermore, sIL-2R level seems to be a good discriminator between CRPS patients and healthy controls. CRPS is still a clinical diagnosis. Until now, no diagnostic test exists for monitoring inflammatory disease activity in CRPS. Based on our findings, sIL-2R could be a promising new marker to determine inflammatory disease activity in CRPS. However, this must be validated in future research.

## Figures and Tables

**Table 1:** Characteristics of the CRPS patients.

Characteristics	*n* = 80
Women (*n*, %)	67 (83.8)
Age in years (mean, SD)	44.4 (12.25)
CRPS duration in months (median, Q3 − Q1)	11 (36 − 5)
Upper limb (*n*, %)	46 (57.5)
*Warm/cold/unknown CRPS*	
Warm CRPS (*n*, %)	30 (37.5)
Cold CRPS (*n*, %)	44 (55.0)
Unknown (*n*, %)	6 (7.5)
*Precipitating injury*	
Trauma (*n*, %)	51 (63.8)
Surgery (*n*, %)	21 (26.3)
Spontaneous onset (*n*, %)	6 (7.5)
Missing (*n*, %)	2 (2.5)

**Table 2:** Results of the binary logistic regression analysis.

		95% CI for odds ratio		
	*B* (SE) [*p* value]	Lower	Odds ratio	Upper
Included				
Constant	−6.96 (1.11) [<0.001]			
sIL-2R	0.002 (<0.001) [<0.001]	1.002	1.002	1.003

*R* ^2^ = .57 (Cox & Schnell), .76 (Nagelkerke), model *χ*^2^_(1)_ = 130.33, *p* < 0.001.
